# Sustainable trap strategies for controlling the dominant waxberry pest *Dicranocephalus wallichii bowringi*: Evidence from three-year field trials

**DOI:** 10.1007/s44297-026-00069-4

**Published:** 2026-03-19

**Authors:** Yu-Xi Zhu, Run Yang, Zhang-Rong Song, Ming‐Hui Gong, Yuan Shen, Zeng Xu, Yu-Zhou Du

**Affiliations:** 1 Department of Entomology, College of Plant Protection, Yangzhou University, Yangzhou, Jiangsu China; 2 Entomology and Nematology Department, University of Florida, Gainesville, FL USA; 3 Bureau of Agriculture and Rural Affairs of Binhu District of Wuxi, Wuxi, Jiangsu China

**Keywords:** Beetle, *Dicranocephalus wallichii bowringi*, Waxberry, Trap, Integrated pest management

## Abstract

Waxberry, a highly valued specialty fruit in China, is frequently threatened by various pests. Among these, certain beetle species, particularly *Dicranocephalus wallichii bowringi*, have emerged as major pests during the fruit harvest season in Jiangsu, China. However, the field occurrence patterns of these beetles remain poorly understood, hindering effective management. Here, we conducted a three-year field study to monitor the population dynamics of beetles in open waxberry orchards and to develop sustainable pest management strategies. Of the three common beetle species identified, *D. wallichii bowringi* was the dominant pest, with peak populations coinciding with fruit ripening. A green trapping device baited with a sugar–vinegar–wine aqueous solution effectively attracted adult *D. wallichii bowringi*. Implementation of this strategy over three consecutive years resulted in marked reductions in *D. wallichii bowringi* populations. These findings offer an eco-friendly and effective trapping strategy for managing this key waxberry pest.

**Supplementary Information:**

The online version contains supplementary material available at 10.1007/s44297-026-00069-4.

## Introduction

**Fig. 1:**
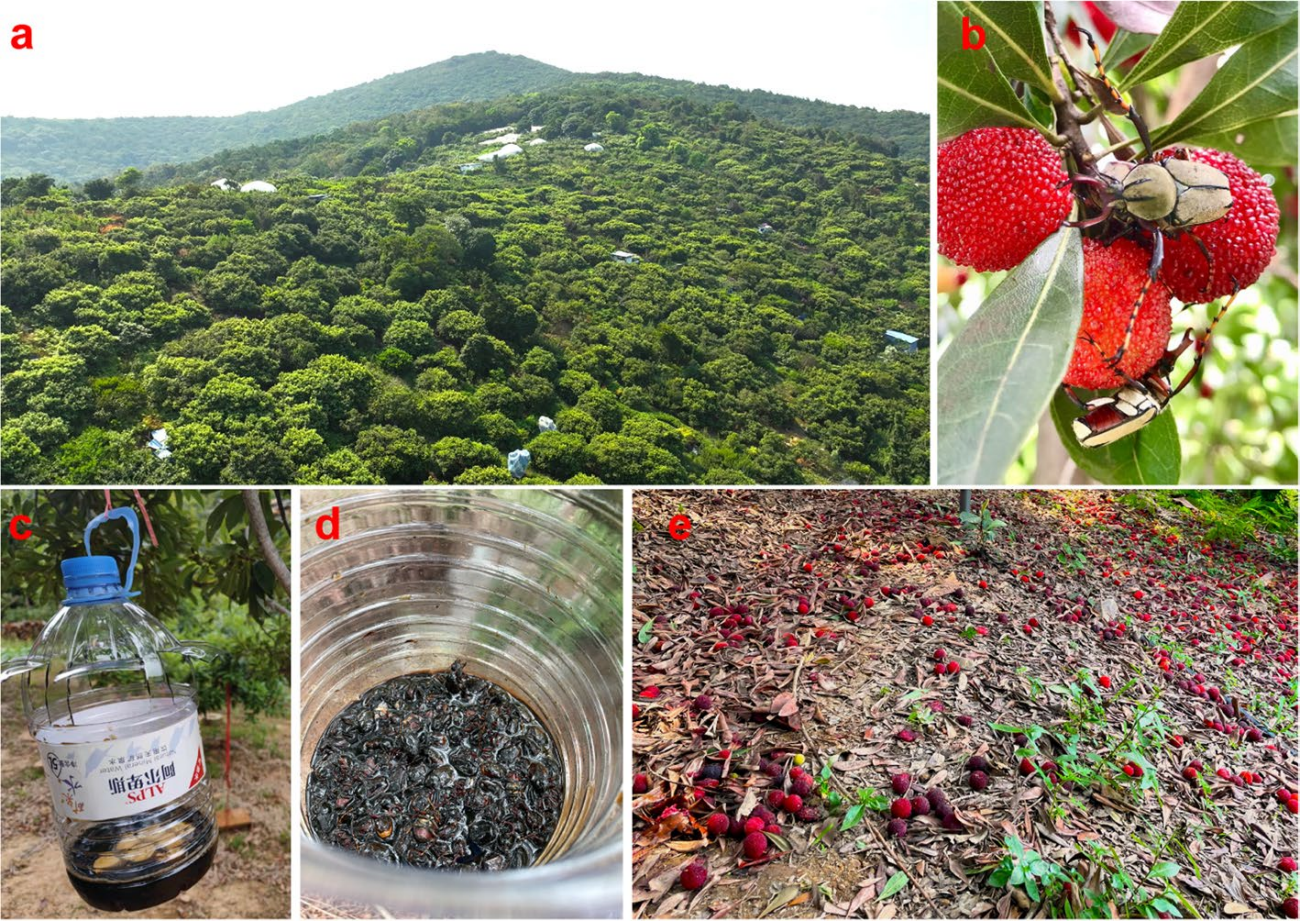
Waxberry orchard and beetle damage to waxberry fruits. **a** Aerial view of the waxberry orchard. **b** The flower beetle *Dicranocephalus wallichii bowringi* feeding on the pulp of a waxberry fruit. **c** Simple beetle trapping device used in the study. **d** Adult beetles captured in the trapping device. **e** Waxberry fruit drop caused by pest infestations in the orchard

Waxberry (*Myrica rubra*, Myricaceae), a highly valued specialty fruit in southeastern China, has experienced rapid cultivation expansion in recent years due to its high nutritional and medicinal value, along with low pesticide residues and increasing consumer demand [1]. However, waxberry fruits are frequently damaged by various insect pests, such as fruit flies and beetles, leading to substantial economic losses despite high yield potential [1, 2]. However, the types of pests and the extent of damage they inflict on waxberry crops vary by region. Notably, our previous studies indicated that since 2019, beetles, particularly *Dicranocephalus wallichii bowringi* (Coleoptera: Cetoniidae), have caused significant damage in Wuxi City, Jiangsu Province, posing a serious threat to the local waxberry industry [2]. These beetles damage the fruit by using their chewing mouthparts to feed on the flesh and their sharp claws to tear the skin, making the fruit more vulnerable to disease and drop [2, 3] (Fig. 1). Thus, a deeper understanding of the field dynamics and damage patterns of beetle pests is critical for developing effective pest control strategies.

Monitoring pest populations is a cornerstone of sustainable pest management [4, 5]. Various pest monitoring methods, including entomological net mowing, window traps, pitfall traps, light traps, and malaise traps, have been widely used in beetle surveillance [6–8]. However, these methods are not always effective for specific pest species and often require significant labor and resources. Within this context, there has been growing interest in developing more sustainable alternatives, including simpler physical trapping strategies, for monitoring and control of beetles [4, 9]. Previous studies have revealed that traps based on light or chemical odors are highly effective for monitoring and controlling various pest species, but their efficacy depends on host species [10–12]. Despite advancements in beetle monitoring, the newly emerged pest *D. wallichii bowringi* presents new demands for understanding its field occurrence patterns and developing eco-friendly and effective management methods [2, 3]. Sole reliance on commercial chemical pesticides poses risks to human health and the environment, especially given their impracticality during the ripening period. This underscores the urgent need for sustainable and targeted management strategies.

In this study, we investigated the species composition, occurrence patterns, and damage caused by pest beetles in waxberry orchards in Wuxi city. We identified the dominant pest species and explored eco-friendly, effective control methods. Our goal was to develop innovative and sustainable strategies for selective trapping, which can be integrated into an integrated pest management approach for open-field waxberry orchards.

## Results

### Field occurrence dynamics of the three beetle species

To monitor beetle populations, we deployed 10 traps (Fig. 1c) in the waxberry orchard and tracked the field occurrence dynamics of three beetle species: *Dicranocephalus wallichii bowringi*, *Protaetia orientalis*, and *P. brevitarsis* (Table S1). All three species exhibited one generation per year in the local environment (Fig. 1).

**Fig. 2:**
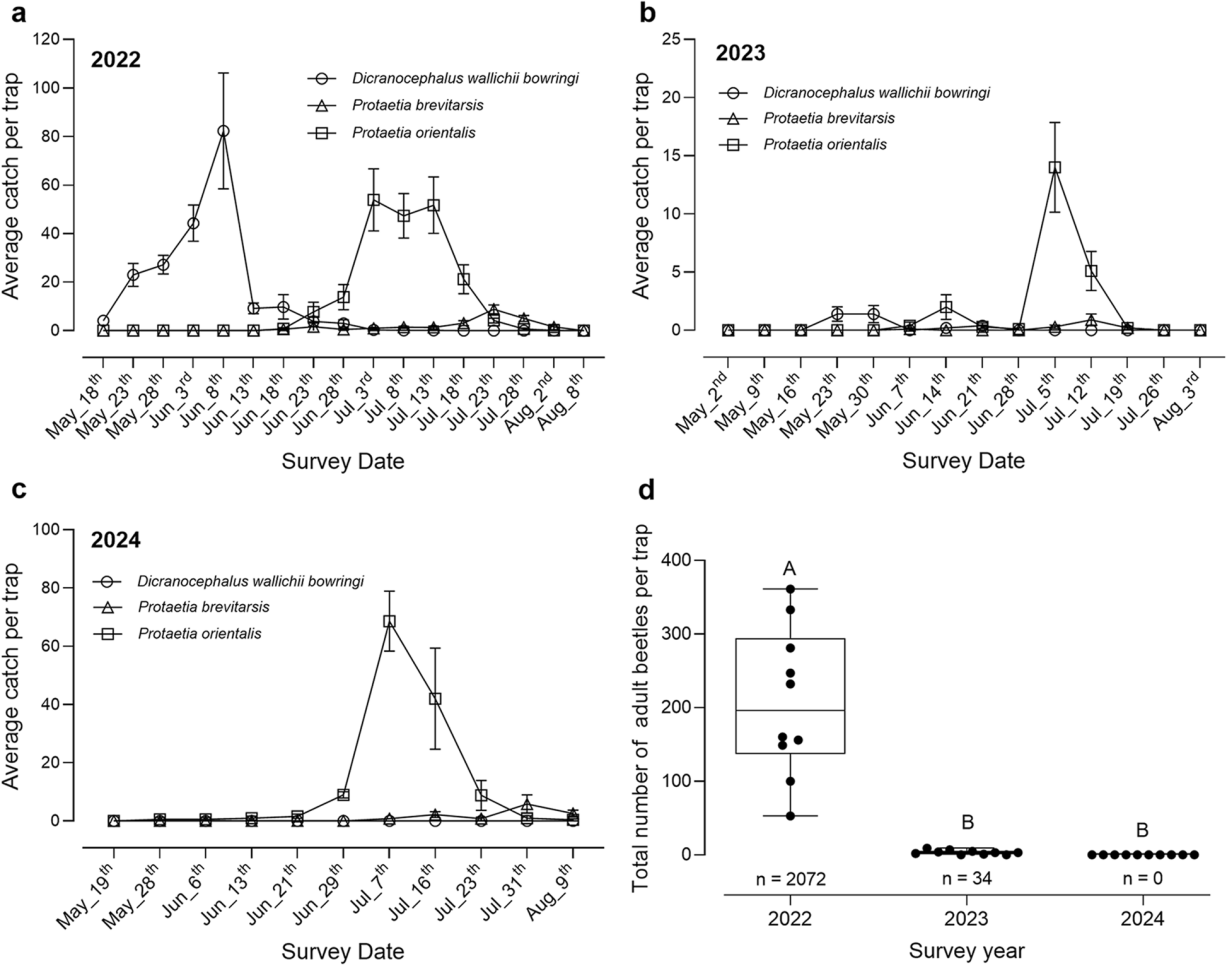
Occurrence dynamics and control effectiveness of beetles in open-field waxberry orchards. Population dynamics of three beetle species in open-field waxberry orchards over three consecutive years: 2022 (**a**), 2023 (**b**) and 2024 (**c**). The data show the average catch number of each beetle species per trap. **d** Evaluation of control effectiveness based on the number of *Dicranocephalus wallichii bowringi* captured in the field. Different capital letters indicate significant differences among groups (*P* < 0.05). *P* values were calculated using Kruskal–Wallis tests

From 2022 to 2024, adult *D. wallichii bowringi* emerged sporadically in early May. In 2022, the average number of beetles captured per trap gradually increased starting on May 18th, peaked on June 8th (~ 82 per trap), and then sharply declined by June 13th (Fig. 2a). Following mass trapping efforts in 2022, only a few *D. wallichii bowringi* individuals were captured in 2023, and none were detected in 2024 (Fig. 2a-c). The occurrence period of *D. wallichii bowringi* coincided with the waxberry fruit harvesting period (mid-May to mid-June), causing significant damage to the fruit.

In contrast, the field occurrence patterns of *P. orientalis* remained consistent from 2022 to 2024 (Fig. 2a-c). We observed a sharp increase in adult *P. orientalis* populations in late June, peaking in early July (Fig. 2). Notably, *P. brevitarsis* maintained relatively low population levels throughout the three-year study period (May 2022 to August 2024) (Fig. 2). The peak activity of these two species occurred after the waxberry harvest, resulting in minimal impact on fruit yield. Collectively, these findings identify *D. wallichii bowringi* as the dominant pest affecting waxberry fruits.

### Effects of attractant solutions and trap color on the capture of the dominant beetle

**Fig. 3:**
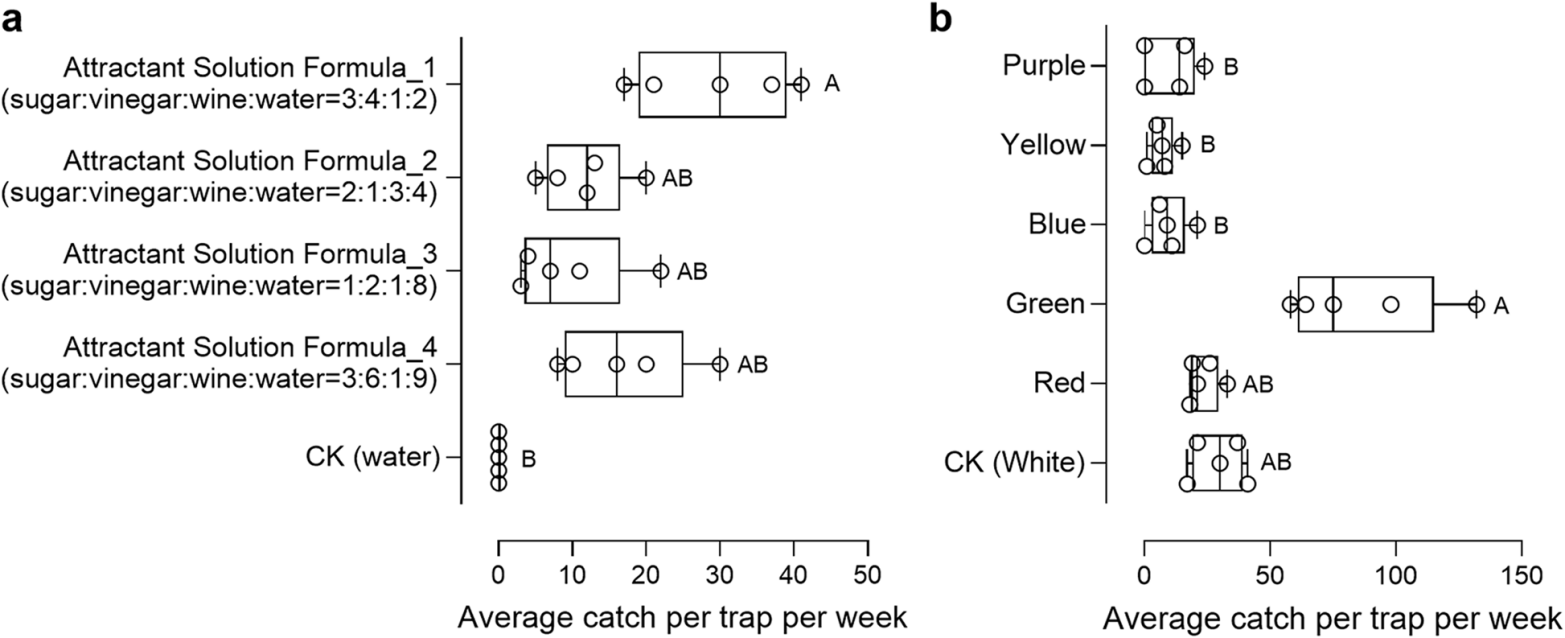
Effects of odor attractants and trap color on adult beetle capture. **a** Average number of *Dicranocephalus wallichii bowringi* captured per trap per week using different attractant solution formulations. **b** Average number of *D. wallichii bowringi* captured per trap per week using traps of different colors. Different capital letters indicate significant differences among groups (*P* < 0.05). *P* values were calculated using Kruskal–Wallis tests

We further examined the effects of attractant solutions and trap color on the capture efficiency of the dominant beetle, *D. wallichii bowringi*, to optimize trapping methods*.* Significant differences in adult beetle captures were observed among odor treatments (Kruskal–Wallis test, *χ*^2^ = 17.45, *p* = 0.0016) (Fig. 3a). Traps baited with water failed to capture any *D. wallichii bowringi* individuals. In contrast, traps baited with Formula 1 (sugar: vinegar: wine: clear water = 3: 4: 1: 2; mean ± SEM: 29.20 ± 4.57) captured more beetles than those baited with Formula 4 (3: 6: 1: 9; 16.80 ± 3.93), Formula 2 (2: 1: 3: 4; 11.60 ± 2.54), or Formula 3 (1: 2: 1: 8; 9.40 ± 3.44), demonstrating a two- to three-fold increase in weekly captures per trap (Fig. 3a).

Additionally, trap bucket color significantly influenced adult *D. wallichii bowringi* captures (Kruskal–Wallis test, *χ*^2^ = 21.83, *p* < 0.001) (Fig. 3b). Green traps captured the highest number of beetles per week (mean ± SEM: 85.40 ± 13.50), followed by white (29.20 ± 4.565), red (23.40 ± 2.768), purple (10.80 ± 4.716), blue (9.40 ± 3.444), and yellow (7.20 ± 2.289) traps (Fig. 3b). Together, these results demonstrate that a green trapping device baited with Formula 1 is highly effective in attracting and killing adult *D. wallichii bowringi*.

### Control efficacy for the dominant beetle D. wallichii bowringi

Significant differences were observed in the total average number of adult *D. wallichii bowringi* captured per trap across the three-year study period (Kruskal–Wallis test, *χ*^2^ = 25.09, *p* < 0.001). In 2022, the average was approximately 207 adult individuals per trap, which decreased approximately 70-fold to approximately 3 individuals per trap in 2023 (Fig. 2d). A total of 2072 adult individuals were captured in 2022, compared to only 34 in 2023. Impressively, *D. wallichii bowringi* was not detected at all in the waxberry orchard in 2024 (Fig. 2d). These results suggest that mass trapping was effective in reducing the *D. wallichii bowringi* population, potentially leading to a population collapse in the orchard.

## Discussion

In this work, we conducted a comprehensive field survey of the occurrence dynamics of three beetle species in Wuxi, with a particular focus on the dominant pest *D. wallichii bowringi*. To our knowledge, this represents the first detailed documentation of its seasonal occurrence patterns, establishing a valuable baseline for generating control strategies. In parallel, we optimized a trapping approach based on a sugar-vinegar-wine mixture, successfully reducing the population of *D. wallichii bowringi* through three years of field mass trapping. The low-cost and easily accessible artisanal traps developed here are readily compatible with integrated pest management programs for waxberry cultivation in open-field orchards.

Understanding the seasonal patterns of pest occurrence is essential for determining the optimal timing for pest management interventions and for designing alternative control strategies [4]. Among the three beetle species surveyed, both *D. wallichii bowringi* and *Protaetia orientalis* exhibited relatively high field densities. However, *D. wallichii bowringi* populations peaked during the waxberry harvest period (mid-May to mid-June), resulting in substantial fruit damage, whereas *P. orientalis* reached peak abundance after harvest and had minimal impact on yield. These findings identify *D. wallichii bowringi* as the dominant pest requiring targeted control measures. The observed dynamics provide a foundation for understanding pest behavior and developing effective management strategies.

Chemical pesticides, while effective for many beetle species [13], are prohibited during the waxberry ripening period to ensure the production of green food. Consequently, physical control strategies, such as shelter traps, light traps, odor attractants, and insect netting, offer simpler and more sustainable alternatives [13–16]. Our preliminary trials indicated that insect netting effectively reduced pest damage but was economically and practically challenging due to the height of waxberry trees (Fig. S1b). Consistent with previous reports showing the effectiveness of sugar–wine–vinegar baits for controlling beetles [15, 17, 18] and flies [19], our field experiments identified this formulation as the most efficient and cost-effective attractant for *D. wallichii bowringi*. Capture efficiency is influenced by multiple factors, including bait composition, trap color and trap placement [20–22]. Environmental conditions, such as temperature, may further modulate attraction by affecting volatile release rates from the bait [23, 24]. Notably, our results suggest that *D. wallichii bowringi* may exhibit enhanced sensitivity to green-colored traps, a hypothesis that warrants further experimental validation. Future studies should also focus on identifying the key volatile compounds responsible for attraction and determining optimal concentrations to maximize trapping efficiency.

Our sugar-vinegar-wine solution trapping method has several advantages compared to other control techniques [13, 25]. It is cost-effective, reducing both material and labor costs. The traps used in this study can be quickly produced using recycled materials or easily accessible items, such as plastic bottles and common food products. The bait components are readily available from grocery stores, making the method simple to implement and easy for fruit farmers to adopt. Moreover, the approach is environmentally friendly, as it reduces reliance on chemical pesticides and promotes the reuse of discarded bottles. This method can significantly decrease pesticide usage, thereby reducing the risks of environmental contamination and pesticide resistance. Additionally, the trapping method is not only effective against *D. wallichii bowringi* but also attracts other pests, such as fruit flies. Given that *D. wallichii bowringi* is a K-strategist pest [26], its populations, once reduced, are unlikely to rebound rapidly and cause a re-infestation. As a result, our control method offers sustainable long-term pest management.

Several limitations of this study warrant further investigation. First, because beetle population dynamics are closely associated with climatic factors such as temperature, humidity and rainfall [16], we cannot entirely exclude the influence of interannual climatic variation on population dynamics, despite relatively minor climatic differences during the three-year survey period. Second, although effective against *D. wallichii bowringi*, the trapping strategy shows limited efficacy against other pests, such as fruit flies, particularly during outbreak periods. Therefore, the occurrence and dynamics of secondary pests in subsequent years should be systematically monitored. Future research should aim to develop more comprehensive pest management strategies with broader applicability. Emerging approaches, including RNAi-based pesticides [27, 28], releases of natural enemies [29], and microbiota-based tools [30–33], offer promising avenues for the development and large-scale implementation of integrated pest management strategies in waxberry orchards.

In conclusion, our three-year field trials reveal that *D. wallichii bowringi* is the dominant beetle pest in waxberry orchards and demonstrate an eco-friendly and effective trapping strategy for its management. This approach provides a practical tool for pest monitoring and regional control. Future work will focus on identifying key attractive volatiles and optimizing the strategy for large-scale implementation in integrated pest management programs.

## Methods

### Experimental sites

Field trials were conducted in an open-field waxberry orchard (cultivar: Biqi, area: approximately 6 ha, age: approximately 15 years) located in Binhu District (120.244°E, 31.496°N; altitude: 62.4 m), Wuxi City, Jiangsu Province, China, from 2021 to 2024 (Fig. 1A). To minimize potential border effects, trials were conducted in the central area of the orchard, covering approximately 0.3 hectares and encompassing 75 trees.

### Preliminary selection of survey methods

To survey beetle pest species in the waxberry orchard, three common methods were employed in 2021: light traps (brand: Jia-neng) (Fig. S1a), sweep nets, and a sugar-vinegar-alcohol solution-based trapping method (Fig. 1). Light traps and sweep nets yielded low beetle captures, while the sugar-vinegar-alcohol solution was highly effective in attracting beetles (Fig. 1). Consequently, we adopted this method for systematic monitoring of beetle field occurrence dynamics and explored its potential as a physical control measure. Beetle species were identified using both morphological examination and PCR-based techniques [3]. Three primary beetle pest species were identified in the orchard (Table S1).

### Monitoring field occurrence of beetles

Adult beetle monitoring was conducted from May to August annually between 2022 and 2024 using a bait mixture consisting of watermelon and a sugar-wine-vinegar solution. Each 1000 mL of attraction bait consisted of 200 g fresh watermelon, 300 g sugar (brand: pure brown sugar), 100 mL wine (Niulanshan aged liquor), 400 mL vinegar (Hengshun white vinegar), and 200 mL water. Traps were constructed from 5000 mL transparent plastic bottles modified with six equidistant 3 cm × 3 cm window openings 18 cm above the base. A 10 cm string was attached to the bottle cap for suspension (Fig. 1). A “Z” shaped sampling pattern was used, and 10 waxberry trees were selected as attractant sampling points. Traps were suspended approximately 1.5 m above the ground at the center of each tree. Traps were inspected approximately every 7 days from May to August each year. During each inspection, trapped beetles were transferred to labeled plastic flasks for subsequent laboratory analysis.

### Optimization of trapping methods for the dominant beetle

To optimize the attraction bait formulation, the following four attractant solutions were evaluated: (i) Attractant Solution Formula 1 (ASF1), sugar: vinegar: wine: clear water = 3: 4: 1: 2; (ii) ASF2, sugar: vinegar: wine: clear water = 2: 1: 3: 4; (iii) ASF3, sugar: vinegar: wine: clear water = 1: 2: 1: 8; (iv) ASF4, sugar: vinegar: wine: clear water = 3: 6: 1: 9; (v) Control (CK), 2000 mL of clear water. Each trap contained 2000 mL of bait and was suspended 1.5 m above the ground. Five replicates were used per treatment, with baits replaced weekly. A five-point sampling method was employed, with traps placed at north, south, east, and west positions on each of five trees. Trap positions were rotated between replicates to eliminate directional bias. Beetle captures were recorded after one week.

To assess whether trap color affects attractiveness, traps of different colors (green, yellow, blue, red, and purple) were tested, each filled with the same attractant solution (Formula 1, sugar: vinegar: wine: water = 3:4:1:2). A white trap was used as the control. All trapping devices of different colors were obtained from local markets. Five trees in the orchard were selected, with traps of different colors suspended at various positions. After one week, the number of beetles captured in each trap was recorded.

### Evaluation of control efficacy for the dominant beetle

To evaluate the efficacy of the sugar-vinegar-wine attractant in controlling the dominant beetle *D. wallichii bowringi* during the ripening period of waxberries, beetle captures were compared in the survey area from 2022 to 2024.

### Statistical analyses

Data are shown as the mean ± SEM. Data were analyzed using Kruskal–Wallis tests. All analyses were conducted using GraphPad Prism version 10.00 (GraphPad Software Inc., San Diego, CA, USA).

## Supplementary Information

Supplementary Material 1: Table S1. Pest species and their associated damage severity in waxberry orchards. Fig. S1. Tools and equipment used for surveying beetle species.

## Data Availability

All data generated or analyzed during this study are included in this published article and its supplementary information files.
