# Supplementary material for: Sustainable trap strategies for controlling the dominant waxberry pest Dicranocephalus wallichii bowringi: Evidence from three-year field trials
Source: Crop Health. 2026 Mar 19;4(1):7. doi: 10.1007/s44297-026-00069-4 (PMC13003048; doi:10.1007/s44297-026-00069-4)
Supplement: Supplementary file 1 — Supplementary Material 1: Table S1. Pest species and their associated damage severity in waxberry orchards. Fig. S1. Tools and equipment used for surveying beetle species. [file 44297_2026_69_MOESM1_ESM.docx]

***Supplementary Materials for***

**Sustainable trap strategies for controlling the dominant waxberry pest *Dicranocephalus wallichii bowringi*: Evidence from three-year field trials**

Yu-Xi Zhu^1^, Run Yang^1^, Zhang-Rong Song^2^, Ming‐Hui Gong^3^, Yuan Shen^3^, Zeng Xu^3^, Yu-Zhou Du^1*^

^1^ Department of Entomology, College of Plant Protection, Yangzhou University, Yangzhou, China

^2^ Entomology and Nematology Department, University of Florida, Gainesville, Florida, USA

^3^ Bureau of Agriculture and Rural Affairs of Binhu District of Wuxi, Wuxi, China

* Corresponding Author: Yu-Zhou Du (yzdu@yzu.edu.cn)

The file includes the following supplementary materials:

Table S1;

Fig. S1

**Table S1. Pest species and their associated damage severity in waxberry orchards.**

| Number | Species | Order | Family | Damage tissue |
| --- | --- | --- | --- | --- |
| 1 | *Dicranocephalus wallichii bowringi* | Coleoptera | Cetoniidae | Fruit |
| 2 | *Protaetia brevitarsis* | Coleoptera | Cetoniidae | Fruit |
| 3 | *Protaetia orientalis* | Coleoptera | Cetoniidae | Fruit |
| 4 | *Anomala corpulenta* | Coleoptera | Rutelidae | Fruit |
| 5 | *Aromia bungii* | Coleoptera | Cerambycidae | Branch |
| 6 | *Drosophila simulans* | Diptera | Drosophilidae | Fruit |
| 7 | *Drosophila suzukii* | Diptera | Drosophilidae | Fruit |
| 8 | *Drosophila melanogaster* | Diptera | Drosophilidae | Fruit |
| 9 | *Jacobiasca formosana* | Homoptera | Cicadellidae | Leaf |
| 10 | *Aleuroplatus pectiniferus* | Homoptera | Aleyrodidae | Leaf |
| 11 | *Amata emma* | Homoptera | Amatidae | Leaf |
| 12 | Adoxophyes cyrtosema | Lepidoptera | Tortricidae | Leaf |
| 13 | Macrotermes barneyi | Blattaria | Termitidae | Root |
| 14 | *Thrips palmi* | Thysanoptera | Thripidae | Fruit |
| 15 | *Gryllotalpa orientalis* | Orthoptera | Gryllotalpidae | Root |


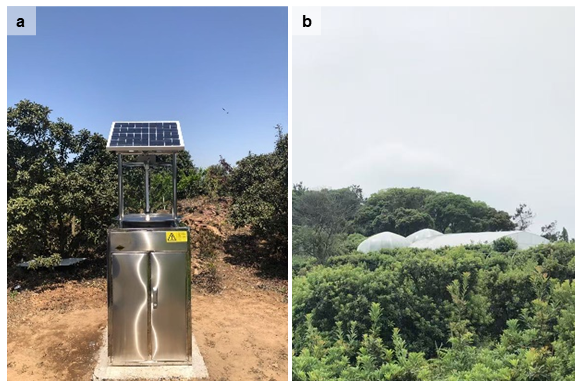


**Fig. S1. Tools and equipment used for surveying beetle species.**
